# Discordantly Low HbA1c in Type 1 Diabetes: Undiagnosed G6PD Deficiency Masking Poor Glycemic Control—A Case Report

**DOI:** 10.1155/crie/3927494

**Published:** 2026-08-27

**Authors:** Javeria Khan, Irfan Khan, Muhammad Maaz bin Zahid, Hoor Qazi, Ayesha Nasir

**Affiliations:** ^1^ Khyber Medical College, University of Peshawar, Peshawar, Khyber Pakhtunkhwa, Pakistan, uop.edu.pk; ^2^ Imperial College London Diabetes and Endocrine Centre, Al Ain, UAE; ^3^ Sardar Begum Dental College, Peshawar, Khyber Pakhtunkhwa, Pakistan

**Keywords:** case report, continuous glucose monitoring, fructosamine, G6PD deficiency, HbA1c, hemolysis, type 1 diabetes mellitus

## Abstract

**Background:**

Glycated hemoglobin (HbA1c) is a widely used biomarker for assessing long‐term glycemic control in patients suffering from diabetes mellitus. However, certain hematologic conditions, such as hemolytic disorders, can produce misleadingly low HbA1c values, potentially masking poor glycemic control and leading to mismanagement of the patient. This case represents a rare but clinically significant scenario, where G6PD deficiency leads to spuriously low HbA1c level, emphasizing the need for alternative glycemic markers.

**Case Presentation:**

We report the case of a 17‐year‐old male who presented with classic symptoms of hyperglycemia and was diagnosed with type 1 diabetes mellitus (T1DM) based on clinical features, elevated blood glucose, high HbA1c (>14%), and positive glutamic acid decarboxylase (GAD) antibodies. One month after initiating basal–bolus insulin therapy, his follow‐up HbA1c unexpectedly dropped to 3%, despite continuous glucose monitoring (CGM) showing incomplete control of glucose level. Work‐up for nonglycemic factors, specifically those affecting the red blood cells (RBCs) revealed normal hemoglobin electrophoresis but confirmed glucose‐6‐phosphate dehydrogenase (G6PD) deficiency. The intravascular hemolysis associated with G6PD deficiency likely shortened RBC lifespan, producing spuriously low HbA1c values. Fructosamine testing was consistent with ongoing hyperglycemia.

**Conclusion:**

This case underscores the importance of considering alternative glycemic markers, such as fructosamine or CGM metrics, when discordance exists between HbA1c and clinical or CGM findings. Early recognition of G6PD deficiency in diabetic patients with such unusual presentation is critical to ensure accurate monitoring and to guide patient education on avoiding oxidative stress triggers.

## 1. Introduction

Glycated hemoglobin (HbA1c) is a widely accepted biomarker for both the diagnosis and monitoring of diabetes mellitus, reflecting average plasma glucose concentrations over the preceding 2–3 months by measuring the proportion of hemoglobin irreversibly bound to glucose [1, 2]. The American Diabetes Association (ADA) and World Health Organization (WHO) recommend HbA1c not only for assessing glycemic control but also as a diagnostic criterion for diabetes [3, 4]. However, HbA1c reliability is predicated on the assumption of a normal erythrocyte lifespan and hemoglobin structure. Conditions that reduce red blood cell (RBC) survival, such as hemolytic anemia, acute blood loss, hemoglobinopathies, or enzymatic deficiencies, may lead to spuriously low HbA1c values, potentially masking poor glycemic control [5–7].

Glucose‐6‐phosphate dehydrogenase (G6PD) deficiency is the most common enzymatic RBC disorder, affecting over 400 million people worldwide, with high prevalence in Africa, the Middle East, and parts of Asia [8, 9]. The deficiency predisposes RBCs to oxidative stress–induced intravascular hemolysis, which shortens the RBC lifespan and can lower HbA1c values independent of glycemia [10, 11]. In patients with diabetes, disparities between HbA1c and actual glycemic status can delay treatment optimization and mislead clinical assessment [12].

We report a case of newly diagnosed type 1 diabetes mellitus (T1DM) in an adolescent male in whom a striking discordance between HbA1c and continuous glucose monitoring (CGM) data led to the diagnosis of an underlying G6PD deficiency. This case highlights the importance of recognizing conditions that confound HbA1c interpretation and supports the use of alternative glycemic markers such as fructosamine or glycated albumin in such scenarios. Our report follows the CARE checklist and is in accordance with the Helsinki Declaration.

## 2. Case Presentation

A 17‐year‐old boy presented to clinic on September 27^th^, 2024, with a 3 months history of polyuria, polydipsia, and polyphagia. He reported an unintentional weight loss of around 10 kg in the past 3 months. On investigation, the patient had hyperglycemia and a raised HbA1c of >14%. Autoantibody testing was positive for glutamic acid decarboxylase (GAD) antibody and negative for IA2. T1DM was diagnosed.

Treatment with a basal bolus insulin regimen (MDI regime ‐Tresiba 20 units once daily + Humalog 16 units three times daily) was started. The patient was educated about diabetes and life style modification. He was called for a follow‐up session next month. At the follow‐up, laboratory testing revealed an HbA1c of 3%—an implausible reduction. It was inconsistent with the severity of his initial presentation and discordant with the kinetics of HbA1c decline. Lab error was ruled out by repeating the test.

A nonglycemic factor like hemolysis or an increase in RBC turn over affecting the HbA1c value was suspected. GMI derived from a CGM device revealed normal or elevated blood glucose levels. This discrepancy further supported the possibility of some sort of hemoglobinopathy. A complete blood count and Hb electrophoresis were done to rule out thalassemia. Both tests were normal. The G6PD assay was done. Test results revealed G6PD deficiency, which explained the discordant HbA1c levels, as the intravascular hemolysis leads to a falsely low HbA1c level by reducing RBC life span. Additional tests like hemoglobin, reticulocyte count, and bilirubin levels were done. Hb was slightly reduced, reticulocyte count was slightly raised and bilirubin was slightly raised, which further supported hemolysis. A fructosamine test was done to assess the glucose value independent of red cell turnover. It was also consistent with the GMI.

## 3. Diagnosis

A diagnosis of G6PD deficiency was made along with T1DM and further tracking of glucose was done by testing for fructosamine and CGM, instead of HbA1c. The new diagnosis was communicated to the patient and family. They were educated about G6PD deficiency anemia and how to avoid oxidative stress.

## 4. Diagnostic Tests

On his first visit, the patient presented with classical symptoms of T1DM. The diagnosis was confirmed with HbA1c and GAD antibodies. On follow‐up next month, the HbA1c was found to be discordantly low, which led to a diagnosis of severe G6PD deficiency, where G6PD was <17.00 U/10^12^ RBC (reference range: 222.00–572.00). G6PD was markedly reduced. Table 1 shows the HbA1c values that were obtained on every visit. Hemoglobin level was slightly reduced, that is, 12.5 g/dL (normal range 13–17 for a 17‐year‐old boy). The bilirubin level was slightly raised, that is, 24 µmol/L (normal range 3.4–20.5 µmol/L). Fructosamine values were also consistent with the GMI, that is, fructosamine = 281 umol/L (normal range = 205–285). Table 2 shows all of these relevant key diagnostic parameters. On further follow‐ups, the patient’s blood glucose was monitored by GMI instead of HbA1c. The CGM metrics across all follow‐up visits are summarized in Table 3.

**Table 1:** HbA1c values on each follow‐up.

Date	HbA1c (%)
September 27, 2024	>14
October 2, 2024	>14
October 29, 2024	3.0
November 28, 2024	5.2
February 25, 2025	4.6
May 27, 2025	5.1

**Table 2:** Key diagnostic laboratory findings and reference ranges.

Parameter	Patient value	Reference range
G6PD level (U/10^12^ RBC)	<17.00	222.00–572.00
Hemoglobin, HB (g/dL)	12.5	13–17
Total bilirubin (µmol/L)	245	3.4–20.5
Fructosamine (umol/L)	281	205–285

**Table 3:** GMI matrices on each follow‐up (device name: FreeStyle LibreLink + 2).

Date	GMI (% or mmol/mol)	Average glucose (mg/dL)	Time in range (%)	Time above range (%)	Time below range (%)	Sensor active (%)
October 29, 2024	6.9% or 52	152	90	27	3	97
November 28, 2024	6.6% or 49	139	85	15	0	96
February 25, 2025	6.4% or 46	128	87	11	2	92
May 27, 2025	6.5% or 48	135	84	14	2	98

## 5. Treatment

On the first visit and diagnosis of T1DM, treatment was started immediately on basal bolus insulin, MDI regime (Tresiba 20 units once daily + Humalog 16 units three times daily). He was taught about basic principles of management of T1DM, avoidance and management of hyper/hypoglycemia, sick day rules, ketone testing, early features of DKA (diabetic keto acidosis) and when to go to ER. Written information (leaflets) and helpful web addresses for self‐education were provided.

On follow‐up when the HbA1c was found to be discordantly low, parents were advised to closely monitor glucose and reduce Insulin doses as appropriate. Meanwhile, workup for G6PD deficiency was carried out. After the diagnosis of G6PD deficiency, the patient was treated with the same therapy, but on further visits, glucose level was monitored by GMI.

## 6. Discussion

This case highlights a very significant diagnostic hazard. A falsely low HbA1c level in a patient with T1DM, despite having elevated blood glucose levels, measured by CGM. It was later found out that the low HbA1c was due to G6PD deficiency, a type of hemolytic anemia which leads to reduced RBC life span and thus a low HbA1c.

HbA1c is formed by the nonenzymatic glycation of hemoglobin, a protein found in RBC. It reflects the average blood glucose level of a person for the last 2–3 months, that is, the average life cycle of a RBC. HbA1c is one of the most commonly done tests for the diagnosis of diabetes [13]. There are certain conditions, such as hemolysis, that can lead to a reduced RBC life span. The HbA1c level in such patients will also decrease despite having normal glucose levels, as there will be less time for Hb to get glycosylated [14].

At presentation, the patient had an HbA1c of >14%. After 1 month of insulin administration, he came for follow‐up, this time with an HbA1c of 3%, despite having high blood glucose levels that were monitored by a CGM. Fructosamine levels were obtained, which were also consistent with the ongoing hyperglycemia. This made us think about some nonglycemic cause of the drop in HbA1c. A complete blood count was done and Hb electrophoresis to rule out thalassemia, as it can lead to a reduction in Hb [15]. It was found out that the patient had G6PD deficiency anemia by performing an enzyme assay, leading to an implausible reduction in HbA1c. G6PD is an enzyme found in RBCs, which provides protection against reactive oxygen species by producing NADPH, crucial for neutralizing harmful radicals. Infections, malaria, certain drugs (like sulfonamides, anti‐retroviral drugs, hydroxyurea, and vitamin C), and excess fava beans are some factors which can lead to worsening of condition by increasing oxidative stress [16].

Previous cases have also reported increased hemolysis in G6PD deficiency due to insulin administration. Govindrajan et al. [17] reports jaundice due to hemolysis in a 10‐year‐old boy with T1DM and G6PD deficiency, 11 days after administration of insulin. It was due to the drop in the glucose level, causing NADPH loss in RBC, making them sensitive to oxidative stress. This aligns with our case, where HbA1c also dropped following insulin administration. Messina et al. [18] reported hemolytic crises in a nonketotic, euglycemic, 8‐year‐old child. They suggested that this was due to relative hypoglycemia following insulin administration [18]. This effect was quantified by Ripoli et al. [19] in their study, stating a 1.3% drop in HbA1c in G6PD deficient type 1 diabetics and 0.3% drop in HbA1c in people with intermediate G6PD level. Furthermore, the ratio of glucose/HbA1c can also predict the G6PD status efficiently in diabetics and can be used for screening [20].

Our approach to this case included fructosamine and GMI, derived from CGM. The fructosamine test tells us about the glucose level over the past 2–3 weeks by estimating the amount of glucose attached to certain proteins in blood. It is used as an adjuvant to HbA1c and in conditions where HbA1c results are not reliable, like in the presence of certain blood disorders. It is not affected by hemolysis [21]. CGM and GMI values are in alignment with fructosamine, further strengthening our point of discordant HbA1c by hemolysis.

This case was further managed by educating the patient about his condition, guiding him about all the necessary life style modifications and avoiding oxidative stresses.

This case highlights the importance of using other tests like fructosamine, glucose/HbA1c ratio and CGM‐derived matrices for the tracking and management of diabetes. HbA1c is one of the most commonly used laboratory tests in this regard but we should be aware of all the factors affecting it while dealing with diabetes patients.

## 7. Conclusion

This case underscores an important diagnostic pitfall: G6PD deficiency and hemolysis, leading to a falsely low HbA1c. We suggest alternative monitoring strategies like GMI and fructosamine in these patients. Educating the patient is very important for long‐term management. Furthermore, the diagnosis of G6PD deficiency in diabetics is also important because some drugs like sulfonylureas, used in the management of diabetes can trigger hemolysis. It will help with safe medication.

## Author Contributions

All authors have contributed to conceptualization, investigation, and data curation by thoroughly studying related articles. All have contributed to writing – original draft and writing – review and editing. Javeria Khan had full access to all of the data in this study and takes complete responsibility for the integrity of data and accuracy of the data analysis.

## Funding

No funding was received for this project.

## Disclosure

All authors have read and approved the final version of the manuscript.

## Consent

Informed written consent was taken from care‐givers prior to the submission of this article.

## Conflicts of Interest

The authors declare no conflicts of interest.

## Supporting Information

Additional supporting information can be found online in the Supporting Information section.

## Supporting information


**Supporting Information** We have attached a Supporting Information file which is the CARE checklist. We have prepared this case in alignment with the CARE checklist to ensure completeness, transparency, and reporting quality.

## Data Availability

The data that support the findings of this study are available upon request from the corresponding author. The data are not publicly available due to privacy or ethical restrictions.
