# Supplementary material for: Discordantly Low HbA1c in Type 1 Diabetes: Undiagnosed G6PD Deficiency Masking Poor Glycemic Control—A Case Report
Source: Case Rep Endocrinol. 2026 Aug 27;2026:3927494. doi: 10.1155/crie/3927494 (PMC13522717; doi:10.1155/crie/3927494)
Supplement: Supplementary file 1 [file CRIE-2026-3927494-s001.docx]

**CARE Checklist**

| **Item** | **Topic** | **Checklist item description** | **Line/page** |
| --- | --- | --- | --- |
| 1 | **Title** | The words “case report” should be in the title along with the area of focus | Page 1, 1^st^ line |
| 2 | **Keywords** | Four to seven key words—include “case report” as one of the key words | Page 1 |
| 3a | **Abstract** | **Background:** What does this case report add to the medical literature? | Page 1 |
| 3b |  | **Case Presentation:** chief complaint, diagnoses, interventions, and outcomes | Page 1 |
| 3c |  | **Conclusion:** What is the main “take-away” message from this case? | Page 1 |
| 4 | **Introduction/background** | The current standard of care and contributions of this case—with references (1-2 paragraphs) | Page 1 and 2 |
| 5 | **Timeline** | Information from this case report organized into a timeline (table or figure) | Page 3 |
| 6a | **Patient Information** | De-identified demographic and other patient or client specific information | Page 2 |
| 6b |  | Chief complaint—what prompted this visit? | Page 2 |
| 6c |  | Relevant history including past interventions and outcomes | Page 2 |
| 7 | **Physical Exam** | Relevant physical examination findings |  |
| 8a | **Diagnostic** | Evaluations such as surveys, laboratory testing, imaging, etc. | Page 3 |
| 8b | **Assessment** | Diagnostic reasoning including other diagnoses considered and challenges | Page 2, 3 |
| 8c |  | Consider tables or figures linking assessment, diagnoses and interventions | Page 3 |
| 8d |  | Prognostic characteristics where applicable | - |
| 9a | **Interventions** | Types such as life-style recommendations, treatments, medications, surgery | Page 3, 4 |
| 9b |  | Intervention administration such as dosage, frequency and duration | Page 4 |
| 9c |  | Note changes in intervention with explanation | Page 4 |
| 9d |  | Other concurrent interventions | Page 4 |
| 10a | **Follow-up and** | Clinician assessment (and patient or client assessed outcomes when appropriate) | Page 4 |
| 10b | **Outcomes** | Important follow-up diagnostic evaluations | Page 4 |
| 10c |  | Assessment of intervention adherence and tolerability, including adverse events | Page 4 |
| 11a | **Discussion** | Strengths and limitations in your approach to this case | Page 4,5 |
| 11b |  | Specify how this case report informs practice or Clinical Practice Guidelines (CPG) | Page 4, 5 |
| 11c |  | How does this case report suggest a testable hypothesis? | Page 4,5 |
| 11d |  | Conclusions and rationale | Page 5 |
| 12 | **Patient Perspective** | When appropriate include the assessment of the patient or client on this episode of care | - |
| 13 | **Informed Consent** | Informed consent from the person who is the subject of this case report | Page 6 |
| 14 | **Additional Information** | Acknowledgement section; Competing Interests (Conflict of Interests statement); IRB approval, (Ethical Committee Approval) when required | Page 5,6 |

**Summary of the case**

| 1 | **Patient (gender, age)** | 17 years, Male |
| --- | --- | --- |
| 2 | **Final Diagnosis** | G6PD deficiency and Type 1 diabetes mellitus |
| 3 | **Symptoms** | Polyuria, polydipsia, weight loss, and HbA1c discordant with blood glucose levels, fructosamine and GMI. |
| 4 | **Medications** | MDI regime (Tresiba 20 units once daily + Humalog 16 units three times daily). |
| 5 | **Clinical Procedure** | No procedure was done, diagnosis was made by laboratory tests and treatment was done by Basal bolus insulin. |
| 6 | **Specialty** | Endocrinology |
